# Musculoskeletal symptoms and their associated risk factors among Saudi office workers: a cross-sectional study

**DOI:** 10.1186/s12891-021-04652-4

**Published:** 2021-09-06

**Authors:** Reem S. AlOmar, Nouf A. AlShamlan, Saad Alawashiz, Yaser Badawood, Badr A. Ghwoidi, Hassan Abugad

**Affiliations:** Department of Family and Community Medicine, College of Medicine, Imam Abdulrahman Bin Faisal University, P.O. Box 1982, Dammam, 32211 Kingdom of Saudi Arabia

**Keywords:** Musculoskeletal disorders, Rapid office strain assessment, Prevalence, Office-workers

## Abstract

**Background:**

Musculoskeletal disorders are common worldwide. Several factors are suggested in their aetiology, one of which is ergonomics alongside other individual factors. This study aims at investigating the prevalence of musculoskeletal disorders among administrative office workers at a large university in Saudi Arabia.

**Methods:**

This cross-sectional study recruited office workers at a Saudi university. A questionnaire was used that involved three sections, the first section consisted of sociodemographic questions, the second included the Rapid Office Strain Assessment (ROSA) checklist to assess ergonomic factors possibly involved, and the third included the Nordic Musculoskeletal Questionnaire to measure the outcome. Bi-variate analyses were performed by Chi-Squared tests and T-tests where appropriate, and a multivariable logistic regression was done to yield odds ratios (OR) and 95% confidence intervals (CIs).

**Results:**

The prevalence of musculoskeletal symptoms in any region during the past 12 months preceding the study was 84.5%, and only 30% have sought medical advice. The most common area of complaint was the lower back (54.5%). After adjustment, age and years of experience were positively associated with musculoskeletal symptoms (OR = 1.04, 95% CI = 1.01–1.09 and OR = 1.10, 95% CI = 1.05–1.15). Normal weight was associated with a significant reduction in risk (OR = 0.10, 95% CI = 0.05–0.18). ROSA score was an independent risk factor (OR = 1.77, 95% CI = 1.05–2.96).

**Conclusions:**

Musculoskeletal symptoms were highly prevalent in the current sample. Identified predictors may support the need for interventions to reduce risk.

## Introduction

Occupational problems may cause considerable suffering and loss of productivity. The Workplace Safety and Health Institute estimated that 160 million people suffer work-related illnesses annually [1]. Between 1996 and 2013, the third largest money expenditure was spent on the treatment of musculoskeletal problems in the neck and lower back with a figure of $87.6 billion [2].

The Centre for Disease Control and Prevention defines musculoskeletal disorders as “injuries of the muscles, nerves, tendons, joints, cartilage, and spinal disc”, [3] and states that work-related musculoskeletal disorders are those in which the work environment contributes to the conditions or worsens it. Musculoskeletal disorders prevalence varies worldwide, and is dependent on the characteristics of the population, type of occupation and the tool used to report the symptoms [4]. In Saudi Arabia, several studies have focused on medical personnel with varying rates. A prevalence of 88.9% was reported were reported for radiologists, [5] and 77.9% among dentists [6]. The very few studies examining musculoskeletal symptoms in office workers did not provide risk estimates, but gave varying prevalence rates ranging between 51 and 70% [7].

Musculoskeletal disorders are multifactorial, where a combination of individual, psychosocial and ergonomic factors are involved. Ergonomics is the scientific discipline that studies the interactions between humans and elements of a certain system. It applies data and methods to enhance well-being and overall system performance [8]. Several studies have proposed a causal relationship between workstation environment, represented by ergonomic factors, and the appearance and/or worsening of musculoskeletal symptoms [9] [10]. Multiple ergonomic factors that are proposed to have a direct causal association involve time spent using the computer, mouse, workstation design and job strain.

Specifically, for computer workstation workers, several instruments are available to assess these biomechanical factors, such as the Rapid Upper Limb Assessment, which assesses postural load of workers and its effects on the neck, trunk and upper extremities, [11] and the Rapid Office Strain Assessment (ROSA) which is more focused on the characteristics of the workplace rather than posture, [12] which gives an overall score. The allows to study the effect of workplace ergonomic and occupational factors on the musculoskeletal symptoms in any body region.

Only a limited amount of research has been done to investigate the effect of ergonomics on musculoskeletal disorders among office workers using the ROSA score. A study in 2015, aimed to evaluate the presence of musculoskeletal disorders among 38 office workers of an insurance company and office ergonomics using the ROSA ergonomic index to design and implement an occupational gym program. The study did not report the overall score individually, but only reported the mean overall score of 3.61 for all study participants [13]. Also, in an Iranian study of 163 office workers in 2018, 15.95% were categorised as high risk and required ergonomic intervention [14]. However, this overall score was not used in the estimation of risk of musculoskeletal disorders.

To date, no study has quantified an overall ergonomic score utilising any of those ergonomic instruments in Saudi Arabia, especially among office workers and examined its association with musculoskeletal disorders. Therefore, this study aims at investigating the prevalence of musculoskeletal disorders among office workers at a large university in the Eastern region of Saudi Arabia, and determine whether individual, health and the ROSA ergonomic index are predictors of these symptoms.

## Methods

### Study design and participants

This cross-sectional study utilised the QuestionPro survey software (Seattle, WA, USA) to collect data of 451 volunteering office workers. The inclusion criteria were both male and female office workers who were aged between 22 to 62 years at Imam Abdulrahman Bin Faisal University, Rakah Campus, Saudi Arabia. The exclusion criteria included a history of trauma, cancer, surgery, infections, or sores in the musculoskeletal system. The online survey format was chosen as it is easily accessible, cost-effective and is an eco-friendly option. The survey took approximately 9 min to complete.

The data was collected by senior medical students who were trained by - and worked with/and under the supervision of - three Occupational Medicine physicians who are also co-authors in this study. The training covered the three parts of the questionnaire, namely the sociodemographic and health related questions, the ROSA tool, and the Nordic Musculoskeletal Questionnaire. A special focus was given for the ROSA ergonomic index as it required observation of the worker while at his/her workstation. The training also included both theoretical and practical applications of the ROSA ergonomic index to ensure accuracy and minimise observer bias.

Considering that the Rakah campus holds 3000 office workers, and assuming a prevalence of musculoskeletal disorders of 74%, [15] the minimum required sample was 400, at an alpha level of 0.05 and 4% margin of error.

Following an ethical approval from the Institutional Review Board of Imam Abdulrahman Bin Faisal University (IRB-2020-01-403), and upon approaching prospective office workers, a brief introduction of the research, its aims, voluntary nature, and assurance of anonymity of the data was explained and an informed consent was given. Due to the fact that the office workers had to be observed at their prospective workstations, a non-probability sampling technique was used to recruit participants. The volunteering participants were given the contact information of the principal investigator and were encouraged to contact her for any questions pertaining to the study. The data collection process commenced between the 10th and the 31st of March 2021.

### Questionnaires

#### Sociodemographic and health related questions

Participants were asked basic sociodemographic questions including, age, gender, marital (Single, married, divorced, widowed) and educational status (High school, bachelor’s degree, Higher education). Other questions regarding work included years of experience, and handedness (Left-handed, right-handed). Health related questions included height, weight, and the presence of chronic conditions.

#### Rapid office strain assessment checklist

The ROSA is a checklist designed to be able to rapidly quantify potential risks that may be associated with computer-based office work [12]. It considers workplace posture as well as an assessment of office equipment. The English original version was used in this study which may be accessed on the following link: http://ergo.human.cornell.edu/ahROSA.html.

The checklist operates by assigning postures that are ideal or neutral to a score of 1, which is the minimum score for each area, and any deviations from this minimum score were scored in a linearly increasing manner from values of 1 to 3. Concurrent factors e.g., chair height is not adjustable, were given a score of + 1, which were subsequently added to the base risk factors. The risk factor areas included the chair, monitor, telephone, keyboard, and mouse. The maximum score that is achieved for all sub-sections are indicative of the presence of all risk factors and the maximum duration of use. In comparing the scores of each subsection with the standard final score charts, the final score which ranges between 1 and 10 is found. The ROSA final scores were found to have high inter- and intra-observer reliability.

Construct validity of the ROSA has identified a value of ≥5 as high risk and a value of < 5 as low risk. This final score was subsequently entered into the online survey and was used as the main independent risk factor in the study.

#### The Nordic musculoskeletal questionnaire

To determine which body regions were affected by musculoskeletal symptoms, the Nordic Musculoskeletal Questionnaire was used [16]. It is a valid and reliable screening tool and includes questions on trouble (ache, pain, or discomfort) during the last 7 days, trouble (ache, pain, or discomfort) during the last 12 months, and whether this trouble has prevented the individual from carrying out normal activities (e.g., housework or hobbies) during the last 12 months. The questions cover nine body regions, namely, the neck, shoulders, elbows, wrists/hands, upper back, lower back, hips/thighs/buttocks, knees, and ankles).

The outcome in this study was the presence of musculoskeletal symptoms in any of the nine body regions that have prevented the individual from carrying out normal activities during the last 12 months. A response of “yes” to any of the nine body regions was coded as a “yes”, whereas a response of “no” to all body regions was coded as a “no”.

### Statistical analysis

Data were analysed using the Stata statistical software V.15 [17]. For descriptive statistics, frequencies and percentages were used for categorical variables, means and standard deviations were used for continuous variables.

The Chi-squared test and Student’s t-test were used to present associations between sociodemographic variables, ROSA ergonomic index score and the presence of musculoskeletal disorders. A *p* value < 0.05 was considered statistically significant. A binary logistic regression analysis was used to present odds ratios (OR) along with their 95% confidence intervals (CIs). Choice of variables to include in the final model was decided based on a Directed Acylic Graph (DAG) of associations between the main risk factor and the outcome and was not solely based on significant *p*-values in the bi-variate analyses. Model diagnostics, including residual analyses, were performed to determine a good model fit.

## Results

### Characteristics of participants

A total of 451 office workers agreed to participate in the study. Overall, there were 55.9% males and 44.1% females. The mean age was 38.63 ± 8.5 years, whilst the mean years of experience was 11.95 ± 8.3 years. Regarding the ROSA ergonomic index score, 33.7% of the participants were characterised as a high risk. Of the total sample, 52.3% reported disabling musculoskeletal disorders in the past year (Table 1).

**Table 1:** Characteristics of participating office workers

Characteristics	Number of participants (%)
**Age*(x̄, SD)***	38.63 (8.5)
**Gender**	
Males	252 (55.9)
Females	199 (44.1)
**Marital status**	
Single	95 (21.1)
Married	347 (76.9)
Divorced	9 (02.0)
**Educational level**	
High school	137 (30.38)
Bachelor’s degree	234 (51.88)
Higher education	80 (17.74)
**BMI**	
Normal weight	155 (34.4)
Overweight	165 (36.6)
Obese	131 (29.0)
**Years of experience*(x̄, SD)***	11.95 (8.3)
**Handedness**	
Left	425 (94.2)
Right	26 (05.8)
**Chronic conditions**	
No	341 (74.6)
Yes	110 (24.4)
**ROSA score**	
Low risk	299 (66.3)
High risk	152 (33.7)
**Disabling musculoskeletal symptoms in the past 12 months**	
Absent	215 (47.7)
Present	236 (52.3)

#### Musculoskeletal symptoms among the participants

Among all participants, 58.5% have reported musculoskeletal symptoms occurring in at least one region of the body during the last 7 days preceding the study. Whilst 84.5% have reported musculoskeletal symptoms during the past year, 52.3% disabling symptoms during the last year and only 30.6% have visited a physician due to a musculoskeletal symptom. The musculoskeletal symptoms were found to vary depending on the affected body region and are presented in Table 2.

**Table 2:** Musculoskeletal symptoms across participating office workers

Body part	In the past 7 days	In the past 12 months	Disabling in the past 12 months	Seen a doctor
**Any part**				
No	187 (41.5)	70 (15.5)	215 (47.7)	313 (69.4)
Yes	264 (58.5)	381 (84.5)	236 (52.3)	138 (30.6)
**Neck**				
No	343 (76.1)	225 (49.9)	373 (82.7)	386 (85.6)
Yes	108 (23.9)	226 (50.1)	78 (17.3)	65 (14.4)
**Shoulder**				
No	309 (68.5)	218 (48.3)	315 (69.8)	383 (84.9)
Yes	142 (31.5)	233 (51.7)	136 (30.2)	68 (15.1)
**Upper Back**				
No	376 (83.3)	276 (61.2)	393 (87.1)	409 (90.7)
Yes	75 (16.6)	175 (38.8)	58 (12.9)	42 (09.3)
**Elbows**				
No	428 (94.9)	399 (88.5)	435 (96.5)	437 (96.9)
Yes	23 (05.1)	52 (11.5)	16 (03.5)	14 (03.1)
**Wrists/Hands**				
No	409 (90.7)	287 (63.4)	417 (92.5)	422 (93.6)
Yes	42 (09.3)	164 (36.6)	34 (07.5)	29 (06.4)
**Lower Back**				
No	358 (79.4)	205 (45.5)	368 (81.6)	380 (84.3)
Yes	93 (20.6)	246 (54.5)	83 (18.4)	71 (15.7)
**Hips/Thighs/Buttocks**				
No	429 (95.1)	359 (79.6)	424 (94.0)	432 (95.8)
Yes	22 (04.9)	92 (20.4)	27 (06.0)	19 (04.2)
**Knees**				
No	409 (90.7)	306 (67.9)	417 (92.5)	422 (93.6)
Yes	42 (09.9)	145 (32.1)	34 (07.5)	29 (06.4)
**Ankles/Feet**				
No	417 (92.5)	354 (78.45)	431 (95.6)	427 (94.7)
Yes	34 (07.5)	97 (21.5)	20 (04.4)	24 (05.3)

Table 3 presents musculoskeletal symptoms and its association with overall ROSA ergonomic index score. Associations were mostly significant in any and across all areas, except for shoulders and wrists/hands in the 7 days preceding the study and the elbows in the 12 months preceding the study.

**Table 3:** Musculoskeletal symptom and their association with ROSA ergonomic index score across participating office workers

Body part	In the past 7 days		In the past 12 months		Disabling in the past 12 months		Seen a doctor	
	Rosa Score		ROSA Score		ROSA Score		ROSA Score	
	Low risk	High risk	Low risk	High risk	Low risk	High risk	Low risk	High risk
**Any part**								
No	147 (78.6)	40 (21.4)	67 (95.7)	3 (04.3)	177 (82.3)	38 (17.7)	228 (72.8)	85 (27.2)
Yes	152 (57.6)	112 (42.4)	232 (60.9)	149 (39.1)	122 (51.7)	114 (48.3)	71 (51.5)	67 (48.5)
*P-value*	**< 0.001**		**< 0.001**		**< 0.001**		**< 0.001**	
**Neck**								
No	245 (71.4)	98 (28.6)	162 (72.0)	63 (28.0)	263 (70.5)	110 (29.5)	264 (68.4)	122 (31.6)
Yes	54 (50.0)	54 (50.0)	137 (60.6)	89 (39.4)	36 (46.1)	42 (53.9)	35 (53.8)	30 (46.2)
*P-value*	**< 0.001**		**0.01**		**< 0.001**		**0.02**	
**Shoulder**								
No	212 (68.6)	97 (31.4)	163 (74.8)	55 (25.2)	224 (71.1)	91 (28.9)	267 (69.7)	116 (30.3)
Yes	87 (61.3)	55 (38.7)	136 (58.4)	97 (41.6)	75 (55.1)	61 (44.9)	32 (47.1)	36 (52.9)
*P-value*	0.12		**< 0.001**		**< 0.001**		**< 0.001**	
**Upper Back**								
No	259 (68.9)	117 (31.1)	199 (72.1)	77 (27.9)	271 (69.0)	122 (31.0)	278 (68.0)	131 (32.0)
Yes	40 (53.3)	35 (46.7)	100 (57.1)	75 (42.9)	28 (48.3)	30 (51.7)	21 (50.0)	21 (50.0)
*P-value*	**0.009**		**< 0.001**		**0.002**		**0.02**	
**Elbows**								
No	288 (67.3)	140 (32.7)	265 (66.4)	134 (33.6)	294 (67.6)	141 (32.4)	292 (66.8)	145 (33.2)
Yes	11 (47.8)	12 (52.2)	34 (65.4)	18 (34.6)	5 (31.3)	11 (68.7)	7 (50.0)	7 (50.0)
*P-value*	0.05		0.88		**0.003**		0.19	
**Wrists/Hands**								
No	279 (67.5)	133 (32.5)	206 (71.8)	81 (28.2)	285 (68.4)	132 (31.6)	286 (67.8)	136 (32.2)
Yes	23 (54.8)	19 (45.2)	93 (56.7)	71 (43.3)	14 (41.2)	20 (58.8)	13 (44.8)	16 (55.2)
*P-value*	0.09		**< 0.001**		**0.001**		**0.01**	
**Lower Back**								
No	253 (70.7)	105 (29.3)	157 (76.6)	48 (23.4)	268 (72.8)	100 (27.2)	259 (68.2)	121 (31.8)
Yes	46 (49.5)	47 (50.5)	142 (57.7)	104 (42.3)	31 (37.3)	52 (62.7)	40 (56.3)	31 (43.7)
*P-value*	**< 0.001**		**< 0.001**		**< 0.001**		**0.05**	
**Hips/Thighs/Buttocks**								
No	288 (67.1)	141 (32.9)	247 (68.8)	112 (31.2)	292 (68.9)	132 (31.1)	289 (66.9)	143 (33.1)
Yes	11 (50.0)	11 (50.0)	52 (56.5)	40 (43.48)	7 (25.9)	20 (74.1)	10 (52.6)	9 (47.4)
*P-value*	0.09		**0.02**		**< 0.001**		0.19	
**Knees**								
No	279 (68.2)	130 (31.8)	219 (71.6)	87 (28.4)	292 (70.0)	125 (23.0)	289 (68.5)	133 (31.5)
Yes	20 (47.6)	22 (52.4)	80 (55.2)	65 (44.8)	7 (20.6)	27 (79.4)	10 (34.5)	19 (65.5)
*P-value*	**0.007**		**< 0.001**		**< 0.001**		**< 0.001**	
**Ankles/Feet**								
No	280 (67.1)	137 (32.9)	245 (69.2)	109 (30.8)	292 (67.7)	139 (32.3)	286 (67.0)	141 (33.0)
Yes	19 (55.9)	15 (44.1)	54 (55.7)	43 (44.3)	7 (35.0)	13 (65.0)	13 (54.2)	11 (45.8)
*P-value*	0.18		**0.01**		**0.002**		0.19	

*P-values in bold are significant <  0.05*

**Fig. 1:**
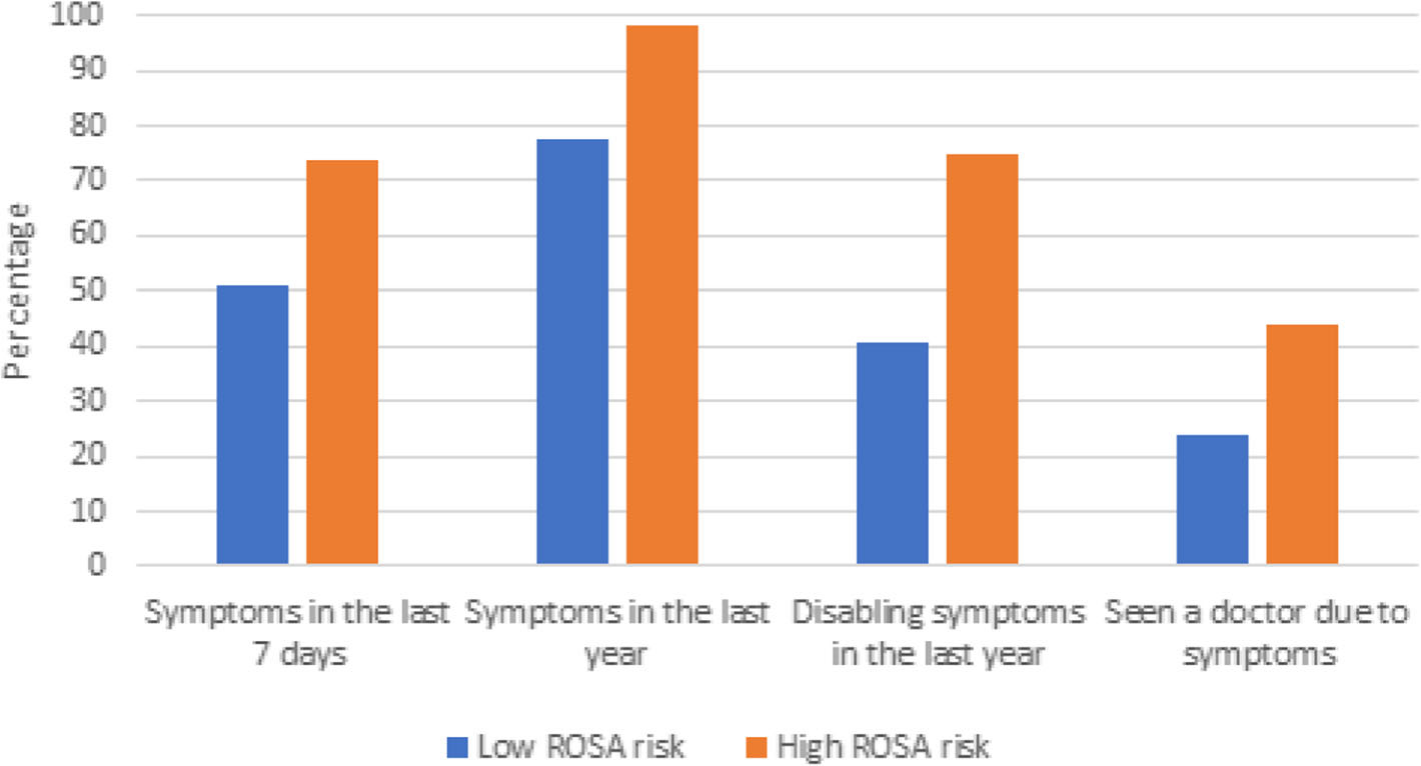
Musculoskeletal symptoms reported in any body region and ROSA ergonomic index score. *Association is highly significant < 0.001*

Figure 1 shows the association between the ROSA scores and the different musculoskeletal symptoms measured. Among all four musculoskeletal outcomes, the majority of all participants were categorised as high risk.

#### Factors related to musculoskeletal symptoms

Musculoskeletal symptoms that have disabled and limited a participant from performing daily normal activities were significantly associated with several factors (Table 4). Older aged participants were significantly associated with higher odds of disabling symptoms (Adjusted OR = 1.04, 95% CI = 1.01–1.09). A similar association was also observed for increased years of experience (OR = 1.10, 95% CI = 1.05–1.15). The results have also shown that being within the normal BMI category was associated with a 90% decreased risk of musculoskeletal symptoms (OR = 0.10, 95% CI = 0.05–0.18) when compared to the overweight category. Although the obese participants were at an increased risk, the association was not statistically significant (OR = 1.09, 95% CI = 0.62–1.92). Furthermore, a high risk of ROSA was significantly associated with an increased odd of disabling musculoskeletal symptoms (OR = 1.77, 95% CI = 1.05–2.96).

**Table 4:** Univariate and multivariable logistic regression analysis

Variables	Disabling in the last year (***N*** = 236)	Univariate			Multivariable	
		X^**2**^ / ***t***	P-value	Unadjusted OR (CI)	Adjusted P-value*	Adjusted OR (CI)**
**Age*(x̄, SD)***	41.8 (07.9)	- 9.0	< 0.001	1.11 (1.08–1.14)	0.04	1.04 (1.01–1.09)
**Gender**		7.2	0.007			
Males	146 (61.9)			1	1	1
Females	90 (38.1)			0.59 (0.41–0.87)	0.18	1.40 (0.84–2.3)
**Marital status**		30.3	< 0.001			
Single	26 (11.0)			0.26 (0.16–0.43)	0.93	0.97 (0.47–1.97)
Married	204 (86.4)			1	1	1
Divorced	06 (02.06)			1.40 (0.34–5.69)	0.43	1.99 (0.35–11.33)
**Educational level**		0.08	0.95			
High school	73 (30.9)			1.06 (0.69–1.62)	0.21	0.70 (0.39–1.23)
Bachelor’s degree	121 (51.3)			1	1	1
Higher education	42 (17.8)			1.03 (0.62–1.71)	0.34	0.71 (0.35–1.44)
**BMI**		116.48	< 0.001			
Normal weight	27 (11.4)			0.09 (0.05–0.16)	< 0.001	0.10 (0.05–0.18)
Overweight	112 (47.5)			1	1	1
Obese	97 (41.1)			1.35 (0.81–2.24)	0.74	1.09 (0.62–1.92)
**Years on the job*(x̄, SD)***	15.30 (0.53)	−9.8	< 0.001	1.13 (1.10–1.17)	< 0.001	1.10 (1.05–1.15)
**Handedness**		0.31	0.57			
Left	15 (06.4)			1.25 (0.56–2.80)	0.93	2.03 (0.66–1.97)
Right	221 (93.6)			1	1	1
**Chronic conditions**		3.4	0.06			
No	170 (72.0)			1	1	1
Yes	66 (28.0)			1.51 (0.97–2.33)	0.21	0.69 (0.38–1.25)
**ROSA score**		47.24	< 0.001			
Low risk	122 (51.69)			1	1	1
High risk	114 (48.31)			4.35 (2.82–6.71)	0.02	1.77 (1.05–2.96)

^* **^Based on logistic regression adjusting for age, sex, BMI, Years on the job and ROSA score

## Discussion

This study has reported an extremely high prevalence of 84.5% of musculoskeletal symptoms among the examined office workers within the last 12 months preceding the study. Over half the participants have reported musculoskeletal symptoms in the past 7 days, as well as disabling symptoms in the past 12 months. In contrast, only 30% have visited a doctor with regards to this issue. This study has also identified that age, BMI, years of experience and ROSA ergonomic index score were significant predictors of disabling musculoskeletal symptoms.

Different figures have been reported both in Saudi Arabia and elsewhere. Among Saudi health professionals, a prevalence of 77.9 and 88.9% were reported for dentists and radiologists within the past 12 months of those respective studies, [5, 6] while a lower prevalence was seen for office workers examined from three major companies ranging between 39 and 51% [7]. Globally, prevalence figures ranged between 74.7% for health occupations, and 80% for office workers [18, 19]. It is important to note however, that although the same Standardised Nordic Questionnaire was used, the referencing symptoms may have been different. This study examined aches, pain, and discomfort symptoms only, whilst others may have investigated other symptoms such as numbness and stiffness.

The most reported musculoskeletal symptoms within the past 7 days preceding this study were in the shoulders, and over half the participating office workers reported symptoms in that region during the past 12 months whilst only 17.3% reported those symptoms as disabling. Researchers have suggested multifactorial models to describe the aetiology underlying shoulder pain [20]. The proposed factors include individual, occupational, and psychosocial work environmental factors. The latter playing an important role in the development and maintenance of chronic pain, where a poor social work environment and the inadequacy to cope with them may cause work-related stress [20]. An increase in stress may increase the relation between physical workload and musculoskeletal symptoms. Stress, however, was not measured in this study.

The lower back region was the most reported area suffering from musculoskeletal symptoms in the past 12 months preceding the study (54.5%). Similar high figures were reported elsewhere, although not as high as the current study [7]. In fact, the prevalence of symptoms in the lower back region reported here is higher than the global prevalence of lower back pain reported in a systematic review of over 160 studies, which found a one-year mean prevalence of only 38% [21]. Like musculoskeletal symptoms in the shoulders, lower back pain aetiology is assumed to be multifactorial in nature. In sedentary occupations that involve prolonged sitting and computer use, sustained lumbar flexion may occur, which is found to limit the spine’s ability to resist force acting upon it, hence exposing the lumbar spine to injury [22].

Despite the high prevalence, only a small portion of the sampled office workers have sought medical advice. None of the local studies have given rates on seeking medical advice, [5–7] and worldwide rates are significantly higher than currently reported in this sample [23]. A possible contributing factor is that those rates come from samples with healthcare related occupations and have medical background knowledge, hence are educated to the possible benefits of treatment. Also, sociocultural differences may have inherently played a role in the hesitancy of the sampled office workers to seek medical advice. Several researchers from a national multistage survey data have observed that despite the extent and density of healthcare facilities and the free healthcare system within the country, Saudis’ abstain from seeking advice unless disease symptoms worsen or have reached advanced stages of illness [24, 25]. Furthermore, there is a high prevalence of the use of complementary and alternative medicine among the Saudi population ranging between 65 and 80% [26], and musculoskeletal symptoms were the most reported reasons for use [27]. It is also possible that workers believe that their musculoskeletal symptoms are related to stress, rather than actual physical causes. Such a belief may have led them to attempt self-treatment although this was not explored.

Age and years of experience were significant predictors. Similar results have been reported in other office workers in Saudi Arabia and elsewhere [7] [28]. Age-related degenerative changes and decreased functional capacity in older workers may have contributed to this association. Furthermore, chronic musculoskeletal fatigue may subsequently cause accumulated stress on muscles and tendons, hence a reduction in blood flow to corresponding areas as work experience increases.

The Global Burden of Disease Study in 2016 stated that obesity and occupational factors are associated with an increased prevalence of musculoskeletal symptoms, more specifically, lower back pain, [29] both of which were also significant predictors in this study. With regards to BMI, a 90% reduction in risk of musculoskeletal symptoms among those who are normal weight is reported here. A direct relationship between the two was reported in multiple studies and confirmed in a systematic review that examined body fat specifically [30]. It is proposed that obesity may cause biomechanical stress on weight bearing joints, where excess loading may change an individual’s gait and posture, hence creating a detrimental biomechanical environment [31].

Ergonomic factors were examined by using the ROSA ergonomic index score. The ROSA checklist, from which the score is derived, has the advantage of considering several factors inherent to a workstation and the ability to combine it to an overall high risk/low risk variable. Office workers with a high-risk score were statistically significantly more likely to report musculoskeletal symptoms. Similar results were reported; however, the ergonomic indices were given for specific areas rather than analysing the overall score [32]. Occupational and ergonomic factors of a workstation including chair height, depth, arm rest, back support, duration, height of the monitor, mouse, keyboard, and telephone usage are all embedded within the ROSA ergonomic index score [12]. In a review of reviews on occupational health and safety interventions that and identified a range of interventions to prevent musculoskeletal symptoms included physical exercise at the workplace, promoting a positive psychosocial working environment, educational and ergonomic interventions within the workstation. They concluded that training alone had no effect on the reduction of those symptoms, whereas a combination of measures is most effective [4].

This study is the first to utilise the ROSA ergonomic index score and draw estimates of risk relating to musculoskeletal symptoms among office-workers in Saudi Arabia. However, it does have certain limitations. First, the sampling design used is a non-probability sampling technique, although this was not intentioned at first. The overwhelming resistance of workers to be observed while working forced the authors to approach workers on a one-by-one basis and ask them to volunteer. Hence, selection bias may have been introduced to the study where those who are more likely to have musculoskeletal symptoms were more likely to agree to participate, especially since the musculoskeletal symptoms are self-reported, which may also be a limitation. Observer bias may have also been introduced, although every effort was made to minimise it through an intensive hands-on training by occupational medicine physicians. It is important to note, that although the prevalence of musculoskeletal symptoms reported was high, occupational and ergonomic factors are not the only contributing factors. Therefore, tt was stressed upon the participating workers to report only work-related symptoms. Lastly, psychosocial factors were not considered.

The extremely high prevalence of musculoskeletal symptoms in this sample merits the consideration of implementing preventive interventions. Singular interventions, such as education only, have not had a significant effect on the reduction symptoms in previous research. A set of interventions which include education, physical activity in the workplace, ergonomic interventions such as adjustable workstations and promoting a positive work environment to reduce work-related stress are highly recommended. Furthermore, we recommend setting up annual check-ups with occupational physicians.

## Conclusion

Musculoskeletal symptoms are highly prevalent among office workers, with lower back pain and shoulder pain being the most frequently reported areas of complaints. Older aged workers, those with more years of experience, overweight, and with high-risk ergonomic scores were associated with a higher rate of musculoskeletal symptoms. The results of this study may be used to develop strategies and interventions to reduce incidences of musculoskeletal symptoms among office workers.

## Data Availability

The datasets used and/or analysed during the current study are available from the corresponding author on reasonable request.
